# The disproportionate burden of pulmonary arterial hypertension among the elderly: global, regional, and national trends from 1990 to 2021—findings from the 2021 global burden of disease study

**DOI:** 10.3389/fcvm.2025.1564785

**Published:** 2025-07-08

**Authors:** Meng Tang, Xingxing Liu, Xiaoyu Hao, Zhiming Liu, Zuwei Li, Hanbin Luo, Guohui Zou

**Affiliations:** ^1^ Jiangxi University of Chinese Medicine, Nanchang, China; ^2^ Guanganmen Hospital, Affiliated to China Academy of Chinese Medical Sciences, Beijing, China; ^3^ Bao'an Authentic TCM Therapy Hospital, Shenzhen, Guangdong, China; ^4^ Jiangxi Province Hospital of Integrated Chinese and Western Medicine, Nanchang, Jiangxi, China; ^5^ The Affiliated Hospital of Jiangxi University of Chinese Medicine, Nanchang, Jiangxi, China

**Keywords:** pulmonary arterial hypertension, prevalence, mortality, disability-adjusted life years, epidemiology, global burden of disease, elderly, aging

## Abstract

**Objectives:**

Pulmonary arterial hypertension (PAH) is a chronic vascular disorder characterized by elevated pulmonary artery pressure and pulmonary vascular resistance, leading to right ventricular failure. This condition imposes a substantial economic burden and significant health challenges globally. This study aimed to comprehensively analyze the global burden of PAH, with specific attention to vulnerable populations including the elderly, by evaluating gender, age (especially older age groups), region, country, and sociodemographic variables using data from the Global Burden of Disease Study (GBD) 2021.

**Methods:**

Utilizing GBD 2021 data, we assessed the global, regional, and national burden and trends of PAH through metrics like age-standardized prevalence, mortality, and disability-adjusted life years (DALYs). Analyses included decomposition, health inequality, and frontier analysis. Age-period-cohort (APC) modeling was used to examine period and cohort effects (1990–2021), and future burden was projected using Bayesian APC (BAPC) modeling.

**Results:**

In 2021, while PAH prevalence and mortality counts were higher than previous years, age-standardized rates (prevalence—ASPR, mortality—ASMR/ASDR) and DALYs showed a declining trend. Crucially, the disease burden was significantly higher among females and, notably, the elderly population compared to males and younger age groups. Low Socio-Demographic Index (SDI) regions consistently bore a disproportionately higher burden of mortality and DALYs. Decomposition analysis identified population growth and population aging as major drivers of the overall PAH burden. Age-period-cohort effects confirmed that disease risk increased substantially with advancing age. BAPC projections suggest a potential decrease in the global PAH burden by 2035.

**Conclusions:**

Progress in reducing the PAH disease burden during 1990–2021 was limited globally, nationally, and regionally. This lack of progress was particularly pronounced among women and, critically, older age groups. Diagnosis, treatment, and prevention strategies remain critically insufficient in low- and middle-income countries. Therefore, proactively developing health policies tailored to the PAH disease burden—with specific consideration for the growing elderly population and aligned with national/regional economic development—is essential to address this major public health challenge.

## Introduction

Pulmonary arterial hypertension (PAH) is a pathophysiological syndrome characterized by the remodeling of the pulmonary vasculature, leading to increased pulmonary vascular resistance and pulmonary arterial pressure, followed by right heart failure and, ultimately, death (1, 2). PAH is a rare yet severe condition that has become a significant and thought-provoking global health issue (3). Studies have indicated (4) that the prevalence of PAH is approximately 1% of the global population, and despite the availability of targeted therapies, the overall 5-year survival rate remains below 60% (4). Furthermore, the pathogenesis of PAH is highly complex (5, 6) and frequently overlaps with a range of other diseases, often resulting in delayed or misdiagnosis. This imposes substantial economic, emotional, and social burdens on patients and healthcare systems alike (7, 8).

The World Health Organization (WHO) classifies pulmonary arterial hypertension (PAH) as Group 1 within the classification of pulmonary hypertension (PH) (9), which includes disorders associated with congenital heart disease, connective tissue diseases, human immunodeficiency virus, portal hypertension, and schistosomiasis (10). Much of the previous research has primarily focused on specific subgroups or other groups of pulmonary hypertension (PH groups 2–5) associated with PAH (11–16), while PAH itself has received relatively limited attention. In recent years, several studies have examined the disease burden of PAH; however, these analyses have often been constrained in both scope and methodology. Emmons-Bell compiled all available epidemiological data on PAH up to 2020 to systematically assess the disease burden, with the primary goal of quantifying the global burden and providing estimation data for the Global Burden of Disease (GBD) PAH epidemiological modeling effort, without offering a detailed interpretation of the findings (17). Similarly, the GBD PAH collaborators reported incidence, mortality, disability-adjusted life years (DALYs), and corresponding age-standardized rates (ASRs) for PAH based on GBD 2021 data. However, this study merely described the disease statistics without conducting an in-depth, comprehensive analysis using novel research methods (7).

In this study, data on pulmonary arterial hypertension (PAH) were obtained from the newly released Global Burden of Disease (GBD) 2021 report, which provided essential baseline information for the analysis. The burden of disease was estimated by stratifying data based on sex, age, and sociodemographic indices, along with age-standardized ratios (ASRs), to gain a clearer understanding of the disease's distribution across different demographic groups. Additionally, innovative research methodologies, including health inequality analysis, decomposition analysis, and frontier analysis, were employed to explore and examine the impact of PAH in greater depth. Temporal, spatial, and future trends of PAH were assessed using various epidemiological indicators, such as the average annual percentage change (AAPC), age-period-cohort modeling, and Bayesian prediction modeling. This study aims to enhance the understanding of the disease burden of PAH and provide a scientific foundation for the rational allocation of healthcare resources. The findings will serve as valuable references and guidance for clinicians, epidemiologists, and policymakers in optimizing healthcare resource distribution and developing more effective public health strategies.

## Material and method

### Data sources

The Global Burden of Disease (GBD) study was initiated in 1991 to provide valid, timely, and critical assessments of health conditions worldwide. It is published biennially, with each iteration updating the available disease data. The GBD 2021 offers a comprehensive epidemiological assessment of 371 diseases, injuries, and 88 risk factors across 21 GBD regions and 204 countries/territories (18, 19). The relevant data are primarily sourced from published systematic reviews, official websites of governmental and international organizations, public bulletins, and demographic and health census reports. The data also benefit from the contributions of GBD collaborators. These datasets encompass a wide range of sources, including censuses, household surveys, civil registration, vital statistics, disease notifications, health service utilization, air pollution monitoring, and satellite imaging (20). For this study, data pertaining to the disease burden of PAH from GBD 2021 were obtained through the Global Health Data Exchange query tool (GHDx, http://ghdx.healthdata.org/gbd-results-tool) and subsequently analyzed as secondary data.

This study used publicly accessible data from GBD 2021, which were de-identified and excluded any personal or sensitive information. Consequently, ethical approval and informed consent were not required. The study was conducted in accordance with the Guidelines for Accurate and Transparent Reporting of Health Estimates (GATHER) (21).

### Definition of disease

The diagnosis of pulmonary arterial hypertension (PAH) was established when the mean pulmonary arterial pressure (mPAP) was ≥25 mmHg, as measured by right heart catheterization (RHC) in the resting supine position. Additionally, PAH was diagnosed when pulmonary capillary wedge pressure (PCWP) was ≤15 mmHg and pulmonary vascular resistance (PVR) exceeded 3 Wood units, in the absence of pulmonary parenchymal or thromboembolic disease (22). In line with the GBD data dictionary, this study adopted the diagnostic codes from the International Classification of Diseases and Injuries (ICD), with PAH being classified under ICD-10 code 127.0 and ICD-9 code 416.0.

### SDI and epidemiologic indicators

The Socio-Demographic Index (SDI) is a composite indicator that reflects the socio-economic development of a geographic area. It is primarily calculated based on three factors: the total fertility rate of women under 25 years of age, the average number of years of schooling for the population aged 15 years and older, and per capita lagged distribution income (23). The SDI ranges from 0 to 1, with 0 representing the lowest level of development and 1 representing the highest level. Based on SDI quintiles, the 204 countries and territories are categorized into five classes: high SDI (>0.81), high-middle SDI (0.70–0.81), middle SDI (0.61–0.69), low-middle SDI (0.46–0.60), and low SDI (<0.46) (24).

The epidemiological indicators emphasized in this study encompassed the age-standardized prevalence rate (ASPR, per 100,000 persons annually), age-standardized mortality rate (ASMR, per 100,000 persons annually), and age-standardized disability-adjusted life years (ASDR, per 100,000 persons annually). These metrics enable comparisons across diverse age cohorts and mitigate the influence of age-structural variations on the outcomes.

### Decomposition analysis

Decomposition Analysis (DA) involves the quantification of population growth, age structure, and epidemiological shifts to elucidate the specific contributions of these factors to trends in prevalence, mortality, and disability-adjusted life years (DALY) associated with disease burden (25). This systematic approach not only aids in revealing the relative impact of individual factors on changes in the burden of pulmonary hypertension, but also facilitates the identification of areas where interventions can be most effectively targeted, thereby enabling the implementation of more focused public health strategies.

### Analysis of health inequalities

This study employed the WHO-recommended Slope Index of Inequality (SII) and Concentration Index (CI) to assess socioeconomically driven cross-national health disparities in the burden of pulmonary arterial hypertension (PAH). The Slope Index represents the slope of the regression line between PAH-related prevalence, mortality, and DALY rates, and the weighted rank of each country and region (26). The Concentration Index is calculated by fitting Lorenz concentration curves to the observed cumulative relative distributions of population, disease prevalence, mortality, and DALY rates, ranked by the Socio-Demographic Index (SDI), and numerically integrating the area under the curve (27).

### Frontier analysis

Frontier analysis refers to the examination of the potential for reducing disease burden based on the socio-demographic development level of each country and region. In this study, the lowest theoretically achievable Age-Standardized Death Rate (ASDR) for pulmonary hypertension was established as a benchmark for optimal performance, reflecting the current level of development in each country and region (28). The disparity between a country or region's current burden and its potential minimum burden was quantified by measuring the absolute distance (i.e., the effective difference) between the 2021 ASDR and the theoretical frontier for each country and region, thereby identifying areas with the greatest potential for improvement.

### Age-period-cohort analysis

We analyzed the effects of age, period, and cohort on the burden of pulmonary arterial hypertension (PAH) by stratifying the Age-Standardized Prevalence Rate (ASPR), Age-Standardized Mortality Rate (ASMR), Age-Standardized Death Rate (ASDR), and demographic data for PAH into six consecutive time periods, ranging from 1992–1996 (median, 1994) to 2017–2021 (median, 2019), and into 20 consecutive cohorts at 5-year intervals (29). Relative risk (RR) represents the ratio between the adjusted factor and the reference factor after accounting for the effects of age, period, and cohort. A RR > 1 indicates that the factor is associated with a higher risk of disease than the reference factor. Net drift refers to the overall annual percentage change in the prevalence, mortality, and DALY rates for PAH, whereas localized drift represents the annual percentage change in these rates for each age cohort, relative to the net drift.

### Bayesian projection modeling

The Bayesian Age-Period-Cohort (BAPC) model has been demonstrated to significantly outperform other linear power models. This model extends the traditional Age-Period-Cohort (APC) framework by incorporating Bayesian statistical methods for probability estimation, thereby providing confidence intervals for predictions and enhancing the accuracy of forecasting PAH disease burden trends through 2035 (30). It has been shown that the probabilistic predictions derived from the BAPC model are well-calibrated, with prediction intervals that are appropriately narrow, avoiding excessive broadness (31).

### Statistical analysis

To assess the temporal trend of age-standardized rates (ASR) for pulmonary arterial hypertension (PAH) from 1990 to 2021, the present study calculated the estimated percentage change (PC), which reflects the relative proportion of change in a given metric (e.g., ASPR, ASMR, and ASDR) over time. The PC is computed as (latest value−initial value)/initial value(\text{latest value} − \text{initial value})/\text{initial value}, multiplied by 100%. The Estimated Annual Percentage Change (EAPC) is used to measure the trend in the change of a specific disease burden or health indicator over consecutive years. In calculating the EAPC, the calendar year was treated as the explanatory variable (*X*), while the natural logarithm of the ASR (ln(ASR)\ln(\text{ASR})) was used as the dependent variable (*Y*). The data were fitted to a regression line *y* = *a* + *bx*+*ɛy* = *a* + *bx* + \epsilon, and the EAPC was calculated using the formula 100 × (exp(*β*) − 1)100\times (\exp(\beta) − 1). The 95% confidence intervals (CIs) were obtained from the linear regression models (32).

The Joinpoint regression model is an analytical approach used to establish segmented regression based on the temporal characteristics of disease distribution. Compared with traditional regression models, the Joinpoint model provides a more detailed assessment of temporal trends in PAH prevalence, mortality, and DALYs across different intervals from 1990 to 2021. The results were expressed as the Average Annual Percentage Change (AAPC). Analyses were performed using the “Segment” and “broomy” R packages, which help identify significant changes in trends over time. The statistical significance of trends was also determined using 95% confidence intervals (CIs) (33).

All statistical analyses and visualizations were conducted using the R statistical software (V.4.3.2). A *p*-value of <0.05 was considered statistically significant. The descriptive analysis in this study primarily aimed at reporting observed numbers and trends, rather than drawing statistical inferences. While changes in trends were observed, caution is advised in interpreting these trends if they do not reach statistical significance.

## Result

### Global burden of disease for PAH

**Figure 1:**
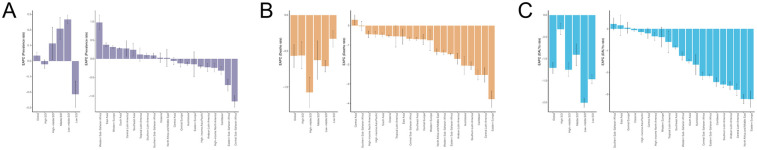
EAPC analysis of PAH **(A)** ASPR; **(B)** ASMR; **(C)** ASDR.

The global prevalence of pulmonary arterial hypertension (PAH) in 2021 was 191,808 individuals (Table 1). Since 1990, there has been a significant overall upward trend, with an 81.46% increase in the number of prevalent cases in 2021 compared to 1990. The Age-Standardized Prevalence Rate (ASPR) for PAH decreased slightly from 2.30 (95% UI: 1.87–2.82) per 100,000 in 1990 to 2.28 (95% UI: 1.85–2.80) per 100,000 in 2021, resulting in an Estimated Annual Percentage Change (EAPC) of 0.03 (95% CI: 0.01 to 0.06) (Figure 1). Regarding mortality, the number of deaths due to PAH increased from 14,842 in 1990 to 22,020 in 2021, representing a 48.36% rise. Concurrently, the Age-Standardized Mortality Rate (ASMR) for PAH decreased from 0.35 (95% UI: 0.29–0.42) per 100,000 in 1990 to 0.27 (95% UI: 0.23–0.32) per 100,000 in 2021, with an EAPC of −0.57 (95% CI: −0.73 to −0.41).The global number of Disability-Adjusted Life Years (DALYs) due to PAH has been declining over the past three decades, reaching 642,104 DALYs in 2021, compared to 1990, a decrease of 6.59%. The Age-Standardized Death Rate (ASDR) for PAH decreased from 13.21 per 100,000 (95% UI: 10.78–15.36) to 8.24 per 100,000 (95% UI: 7.14–9.39), with an EAPC of −1.31 (95% CI: −1.44 to −1.19).

**Table 1:** The case number and ASR of PAH in 1990 and 2021 by SDI quintiles and by GBD regions, with EAPC from 1990 to 2021.

GBD data	Incidence					Deaths					DALYs				
	1990		2021		EAPC (1990–2021) (95% CI)	1990		2021		EAPC (1990–2021) (95% CI)	1990		2021		EAPC (1990–2021) (95% CI)
	All ages (numbers)	Age-standardised rate (per 100,000 people)	All ages (numbers)	Age-standardised rate (per 100,000 people)		All ages (numbers)	Age-standardised rate (per 100,000 people)	All ages (numbers)	Age-standardised rate (per 100,000 people)		All ages (numbers)	Age-standardised rate (per 100,000 people)	All ages (numbers)	Age-standardised rate (per 100,000 people)	
Global															
Both	1,05,703.31 (86,381.45, 130,334.30)	2.30 (1.87, 2.82)	1,91,808.22 (1,55,356.95, 235,787.14)	2.28 (1.85, 2.80)	0.03 (0.01, 0.06)	14,842.49 (12,369.86, 17,484.86)	0.35 (0.29, 0.42)	22,020.53 (18,239.16, 25,351.58)	0.27 (0.23, 0.32)	−0.57 (−0.73, −0.41)	6,87,419.33 (5,35,240.76, 813,086.26)	13.21 (10.78, 15.36)	6,42,104.29 (5,52,272.69, 7,28,993.24)	8.24 (7.14, 9.39)	−1.31 (−1.44, −1.19)
Male	39,421.32 (32,159.43, 48,676.72)	1.75 (1.42, 2.13)	73,126.54 (58,943.78, 89,629.37)	1.78 (1.44, 2.17)	0.15 (0.12, 0.17)	7,326.23 (5,916.90, 8,714.56)	0.37 (0.29, 0.46)	9,578.59 (7,516.26, 11,703.37)	0.27 (0.21, 0.33)	−0.73 (−0.92, −0.54)	3,69,685.76 (3,16,034.69, 4,24,978.10)	14.09 (11.89, 16.35)	2,99,636.21 (2,46,725.64, 3,48,330.88)	8.06 (6.72, 9.36)	−1.54 (−1.69, −1.40)
Female	66,282.00 (54,058.16, 81,489.14)	2.81 (2.29, 3.46)	1,18,681.68 (95,923.29, 1,46,291.08)	2.75 (2.24, 3.39)	−0.02 (−0.04, −0.00)	7,516.26 (5,058.50, 10,160.83)	0.34 (0.23, 0.45)	12,441.95 (10,000.92, 15,375.96)	0.28 (0.22, 0.34)	−0.43 (−0.57, −0.29)	3,17,733.56 (1,94,732.49, 4,44,008.80)	12.32 (7.78, 16.80)	3,42,468.08 (2,82,651.68, 4,30,644.94)	8.39 (6.92, 10.53)	−1.07 (−1.18, −0.96)
SDI quintiles															
High-middle SDI	27,439.19 (22,296.22, 33,800.48)	2.61 (2.12, 3.21)	42,635.88 (34,236.20, 52,931.66)	2.54 (2.07, 3.12)	0.11 (0.01, 0.22)	4,326.29 (3,594.30, 5,141.35)	0.35 (0.31, 0.43)	3,213.79 (2,771.59, 3,908.36)	0.24 (0.20, 0.29)	−1.07 (−1.28, −0.87)	1,27,637.61 (1,06,711.08, 1,54,437.66)	13.14 (10.91, 16.04)	99,448.16 (85,757.43, 1,17,639.39)	6.48 (5.61, 7.87)	−2.20 (−2.36, −2.04)
Middle SDI	29,051.33 (23,769.47, 35,768.60)	2.07 (1.68, 2.54)	59,666.73 (48,064.58, 73,648.38)	2.21 (1.80, 2.71)	0.21 (0.14, 0.28)	7,548.44 (5,141.35, 9,025.54)	0.45 (0.35, 0.58)	4,728.53 (3,774.35, 5,852.09)	0.33 (0.22, 0.39)	−0.63 (−0.89, −0.36)	2,10,945.57 (1,72,856.77, 2,58,349.09)	14.29 (11.66, 17.69)	1,97,170.68 (1,48,781.47, 2,32,321.22)	8.23 (6.26, 9.70)	−1.40 (−1.62, −1.18)
Low-middle SDI	15,078.70 (12,416.61, 18,609.14)	1.77 (1.43, 2.15)	32,714.76 (26,546.45, 40,620.35)	1.90 (1.53, 2.33)	0.27 (0.24, 0.30)	3,727.67 (2,756.51, 5,091.39)	0.35 (0.22, 0.47)	3,124.60 (2,219.57, 3,904.35)	0.26 (0.18, 0.38)	−0.71 (−0.79, −0.63)	1,95,280.82 (1,17,185.14, 2,45,590.74)	14.92 (10.74, 18.41)	1,56,400.32 (1,22,426.28, 1,94,166.50)	9.07 (7.05, 11.60)	−1.33 (−1.43, −1.24)
High SDI	27,053.75 (21,977.20, 33,298.35)	2.67 (2.17, 3.27)	41,452.29 (33,444.95, 51,587.94)	2.64 (2.15, 3.23)	−0.02 (−0.05, 0.01)	4,620.83 (3,919.09, 5,054.49)	0.26 (0.24, 0.28)	2,616.66 (2,379.31, 2,849.99)	0.22 (0.19, 0.23)	−0.56 (−0.75, −0.37)	81,791.97 (77,184.68, 88,110.33)	9.16 (8.71, 9.90)	93,181.57 (84,873.41, 99,191.50)	6.16 (5.76, 6.49)	−1.39 (−1.58, −1.21)
Low SDI	6,965.74 (57,36.97, 8,608.69)	2.08 (1.68, 2.53)	15,180.80 (12,474.43, 18,747.09)	1.94 (1.58, 2.36)	−0.21 (−0.30, −0.13)	1,781.76 (1,146.62, 2,543.99)	0.32 (0.15, 0.51)	1,145.05 (740.49, 1,754.94)	0.27 (0.15, 0.40)	−0.33 (−0.44, −0.22)	71,124.94 (48,628.45, 1,11,613.69)	12.42 (7.78, 19.19)	95,341.84 (67,470.91, 1,33,049.95)	9.30 (6.08, 13.20)	−0.78 (−0.85, −0.70)
GBD regions															
Southeast Asia	6,365.98 (5,233.68, 7,851.60)	1.77 (1.43, 2.17)	13,615.88 (11,019.31, 16,956.99)	1.90 (1.54, 2.32)	0.24 (0.07, 0.41)	741.47 (525.47, 1,849.86)	0.15 (0.09, 0.43)	505.75 (339.93, 1,094.37)	0.12 (0.08, 0.32)	−0.70 (−0.78, −0.61)	27,785.81 (18,241.51, 51,942.76)	6.31 (4.28, 12.72)	31,111.77 (22,912.53, 58,708.38)	4.65 (3.39, 9.25)	−0.92 (−1.01, −0.84)
Central Europe	3,455.75 (2,820.58, 4,227.73)	2.52 (2.08, 3.05)	3,520.66 (2,863.67, 4,311.25)	2.30 (1.89, 2.79)	−0.12 (−0.22, −0.02)	437.61 (397.77, 478.87)	0.26 (0.22, 0.28)	354.96 (309.07, 393.90)	0.21 (0.19, 0.23)	−0.71 (−0.98, −0.44)	11,026.27 (9,783.72, 12,154.07)	8.15 (7.25, 8.95)	10,423.53 (9,511.83, 11,459.38)	6.05 (5.50, 6.67)	−1.01 (−1.26, −0.77)
East Asia	22,867.49 (18,512.91, 28,350.61)	2.07 (1.68, 2.55)	42,486.04 (33,929.79, 53,043.15)	2.23 (1.81, 2.75)	0.38 (0.33, 0.43)	7,489.97 (4,986.46, 9,266.10)	0.59 (0.45, 0.81)	4,114.75 (3,141.43, 5,526.06)	0.41 (0.28, 0.50)	−0.57 (−0.96, −0.17)	1,51,596.32 (1,17,393.64, 2,05,773.17)	15.78 (12.34, 21.21)	1,54,740.44 (1,02,939.01, 1,90,399.49)	8.84 (5.99, 11.01)	−1.32 (−1.69, −0.96)
Oceania	91.52 (74.65, 112.48)	1.98 (1.60, 2.40)	196.27 (158.79, 241.29)	1.80 (1.45, 2.17)	0.03 (−0.10, 0.16)	24.94 (16.69, 43.31)	0.28 (0.18, 0.53)	11.88 (7.61, 19.25)	0.24 (0.16, 0.48)	−0.54 (−0.60, −0.49)	704.47 (448.46, 1,090.11)	10.85 (7.02, 17.55)	1,454.61 (988.20, 2,410.51)	10.14 (6.90, 17.10)	−0.22 (−0.26, −0.17)
Western Europe	15,675.19 (12,581.68, 19,293.62)	3.22 (2.62, 3.95)	23,620.94 (19,120.73, 29,197.29)	3.56 (2.92, 4.35)	0.32 (0.28, 0.35)	1,787.52 (1,533.39, 1,942.67)	0.24 (0.21, 0.27)	1,232.96 (1,093.96, 1,380.14)	0.18 (0.16, 0.19)	−0.76 (−1.28, −0.23)	34,599.00 (31,742.98, 38,280.87)	8.27 (7.69, 9.18)	34,042.54 (31,024.44, 36,440.02)	5.05 (4.75, 5.32)	−1.55 (−2.01, −1.09)
Central Latin America	3,262.25 (2,678.67, 3,991.26)	2.71 (2.21, 3.30)	8,421.49 (6,855.57, 10,382.57)	3.25 (2.64, 3.99)	0.28 (0.07, 0.49)	201.26 (176.86, 230.33)	0.16 (0.14, 0.19)	179.80 (157.27, 209.53)	0.08 (0.07, 0.10)	−2.54 (−2.90, −2.17)	9,966.21 (8,843.65, 11,854.81)	6.12 (5.40, 7.16)	7,245.55 (6,390.76, 8,406.75)	3.00 (2.63, 3.51)	−2.67 (−3.00, −2.34)
South Asia	12,896.59 (10,582.38, 15,994.26)	1.58 (1.28, 1.93)	29,560.95 (23,835.10, 36,926.63)	1.71 (1.38, 2.09)	0.28 (0.27, 0.30)	3,548.80 (2,321.09, 5,531.78)	0.31 (0.17, 0.50)	2,385.25 (1,502.02, 3,417.93)	0.25 (0.16, 0.42)	−0.48 (−0.59, −0.38)	1,36,086.45 (77,808.07, 1,84,381.01)	12.24 (7.79, 17.12)	1,36,562.72 (97,809.04, 1,89,353.32)	8.54 (6.02, 12.46)	−0.95 (−1.03, −0.87)
Eastern Europe	8,178.82 (6,670.46, 10,049.29)	3.21 (2.65, 3.91)	7,686.23 (6,263.74, 9,489.33)	2.83 (2.32, 3.43)	−0.15 (−0.48, 0.19)	278.32 (257.85, 300.35)	0.24 (0.22, 0.27)	562.86 (511.92, 651.32)	0.09 (0.08, 0.10)	−3.78 (−4.20, −3.36)	20,628.22 (18,780.63, 23,859.55)	9.23 (8.47, 10.49)	8,356.54 (7,760.37, 8,996.01)	3.32 (3.10, 3.56)	−3.99 (−4.41, −3.57)
Australasia	669.77 (543.50, 828.03)	2.99 (2.44, 3.70)	1,166.81 (935.86, 1,444.34)	2.83 (2.29, 3.44)	−0.13 (−0.17, −0.09)	57.71 (49.08, 64.86)	0.21 (0.18, 0.26)	45.48 (39.15, 57.18)	0.11 (0.10, 0.13)	−2.06 (−2.51, −1.60)	1,514.25 (1,337.11, 1,856.25)	7.37 (6.49, 9.06)	1,433.51 (1,305.35, 1,562.91)	3.67 (3.38, 3.99)	−2.33 (−2.82, −1.84)
Central Asia	1,408.73 (1,145.19, 1,726.77)	2.46 (2.01, 3.00)	2,161.86 (1,757.90, 2,676.53)	2.33 (1.90, 2.85)	−0.06 (−0.15, 0.04)	318.52 (260.93, 381.55)	0.40 (0.31, 0.47)	208.15 (162.56, 239.83)	0.41 (0.34, 0.48)	0.30 (0.06, 0.54)	9,071.21 (7,195.10, 10,645.59)	14.14 (11.27, 16.45)	11,618.52 (9,513.94, 14,201.81)	12.91 (10.61, 15.60)	−0.33 (−0.55, −0.10)
Southern Sub-Saharan Africa	795.76 (658.44, 966.58)	2.12 (1.73, 2.56)	1,596.23 (1,287.45, 1,944.80)	2.28 (1.85, 2.76)	0.09 (0.03, 0.16)	71.71 (52.97, 85.71)	0.12 (0.08, 0.17)	42.62 (32.26, 58.42)	0.11 (0.08, 0.13)	−0.03 (−0.27, 0.21)	2,187.75 (1,666.89, 2,874.65)	4.70 (3.65, 6.37)	3,211.87 (2,359.18, 38,91.38)	4.33 (3.21, 5.20)	−0.02 (−0.27, 0.22)
High-income Asia Pacific	6,368.84 (5,211.58, 7,811.87)	3.25 (2.67, 3.97)	9,167.25 (7,427.03, 11,361.78)	3.01 (2.47, 3.68)	−0.20 (−0.24, −0.17)	1,048.79 (825.84, 1,200.54)	0.26 (0.24, 0.27)	433.74 (409.85, 459.25)	0.23 (0.20, 0.26)	−0.46 (−0.60, −0.32)	18,474.26 (17,689.14, 19,467.12)	12.03 (11.43, 12.79)	19,988.36 (17,441.90, 21,997.18)	8.31 (7.74, 8.87)	−1.50 (−1.71, −1.28)
High-income North America	5,984.09 (4,856.05, 7,324.59)	1.87 (1.53, 2.29)	8,625.44 (6,917.78, 10,745.03)	1.73 (1.40, 2.12)	−0.24 (−0.35, −0.13)	1,879.78 (1,619.72, 2,042.97)	0.32 (0.28, 0.35)	1,064.41 (946.58, 1,166.98)	0.29 (0.26, 0.31)	−0.45 (−0.62, −0.28)	30,206.11 (27,736.12, 32,714.69)	10.13 (9.39, 10.99)	38,372.60 (35,059.63, 40,844.36)	7.71 (7.17, 8.18)	−1.14 (−1.33, −0.94)
Caribbean	778.03 (634.80, 953.72)	2.56 (2.08, 3.13)	1,228.93 (987.13, 1,505.96)	2.39 (1.93, 2.93)	−0.32 (−0.43, −0.21)	95.37 (65.56, 129.66)	0.38 (0.28, 0.49)	124.17 (83.37, 168.88)	0.20 (0.13, 0.29)	−2.53 (−2.79, −2.28)	7,398.34 (4,169.76, 11,145.34)	19.72 (11.78, 28.82)	5,071.21 (2,987.61, 7,877.35)	11.73 (6.48, 18.74)	−2.01 (−2.20, −1.82)
Andean Latin America	812.23 (666.88, 996.56)	2.91 (2.35, 3.58)	1,777.16 (1,434.00, 2,193.65)	2.77 (2.24, 3.40)	−0.22 (−0.33, −0.12)	91.30 (71.95, 119.39)	0.28 (0.21, 0.35)	83.53 (56.01, 111.88)	0.16 (0.12, 0.20)	−1.70 (−1.98, −1.43)	5,049.04 (2,977.32, 7,421.86)	11.92 (7.83, 16.43)	3,543.75 (2,795.10, 4,489.99)	5.73 (4.52, 7.28)	−2.11 (−2.36, −1.85)
North Africa and Middle East	4,967.64 (4,055.92, 6,153.23)	2.02 (1.64, 2.49)	11,590.78 (9,389.49, 14,435.62)	2.03 (1.64, 2.49)	0.03 (−0.21, 0.26)	1,895.82 (1,328.35, 2,305.00)	0.77 (0.56, 1.00)	2,141.76 (1,308.85, 2,739.26)	0.44 (0.31, 0.53)	−1.36 (−1.51, −1.20)	1,45,728.05 (74,878.55, 2,04,070.24)	35.84 (21.25, 46.14)	80,752.72 (58,085.82, 98,810.33)	14.81 (10.76, 17.96)	−2.43 (−2.57, −2.28)
Eastern Sub-Saharan Africa	2,874.64 (2,358.67, 3,535.65)	2.40 (1.94, 2.92)	5,907.48 (4,822.15, 7,343.53)	2.09 (1.70, 2.53)	−0.71 (−0.87, −0.54)	467.75 (219.41, 877.96)	0.27 (0.12, 0.52)	365.55 (216.88, 686.71)	0.18 (0.07, 0.34)	−1.46 (−1.51, −1.42)	23,147.66 (14,694.41, 45,765.25)	11.11 (6.18, 21.17)	26,604.77 (14,265.05, 49,615.49)	6.85 (3.30, 12.62)	−1.62 (−1.67, −1.58)
Tropical Latin America	2,885.30 (2,356.80, 3,541.16)	2.35 (1.90, 2.88)	6,238.60 (5,019.81, 7,679.45)	2.48 (2.00, 3.04)	0.11 (−0.06, 0.29)	779.39 (713.74, 822.48)	0.37 (0.35, 0.39)	394.12 (372.80, 412.48)	0.32 (0.29, 0.34)	−0.55 (−1.10, 0.00)	19,064.93 (17,856.02, 20,357.09)	14.18 (13.35, 14.98)	24,234.95 (23,001.96, 25,403.31)	10.22 (9.65, 10.78)	−1.12 (−1.68, −0.56)
Western Sub-Saharan Africa	3,111.80 (2,565.50, 3,828.73)	2.42 (1.96, 2.97)	9,318.55 (7,635.34, 11,570.12)	2.97 (2.40, 3.63)	0.98 (0.78, 1.18)	523.27 (305.71, 773.92)	0.25 (0.09, 0.51)	334.68 (194.92, 616.46)	0.17 (0.07, 0.28)	−1.38 (−1.50, −1.26)	19,834.31 (13,552.61, 38,853.02)	9.34 (5.22, 16.94)	32,071.03 (22,084.07, 47,221.86)	6.50 (3.77, 9.47)	−1.14 (−1.27, −1.00)
Southern Latin America	1,280.90 (1,039.53, 1,574.37)	2.69 (2.18, 3.30)	2,227.32 (1,800.20, 2,759.99)	2.82 (2.29, 3.48)	0.10 (0.05, 0.15)	150.04 (138.46, 161.77)	0.36 (0.32, 0.40)	169.34 (150.60, 186.22)	0.18 (0.17, 0.20)	−2.06 (−2.26, −1.86)	7,983.40 (7,181.48, 8,803.28)	16.28 (14.65, 17.95)	4,739.32 (4,439.05, 5,091.53)	6.55 (6.13, 7.07)	−2.87 (−3.02, −2.72)
Central Sub-Saharan Africa	971.97 (795.46, 1,205.95)	2.79 (2.26, 3.44)	1,693.37 (1,377.93, 2,093.53)	1.86 (1.49, 2.24)	−1.14 (−1.30, −0.99)	131.19 (61.77, 237.43)	0.24 (0.11, 0.47)	86.74 (54.87, 162.55)	0.19 (0.08, 0.37)	−0.67 (−0.74, −0.60)	5,367.28 (3,258.01, 10,524.66)	9.09 (5.65, 17.13)	6,524.00 (3,586.22, 11,025.25)	6.16 (2.94, 11.09)	−1.14 (−1.24, −1.04)

**Figure 2:**
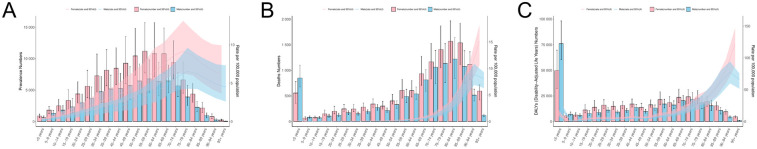
Population analysis of PAH **(A)** ASPR and the number of prevalence; **(B)** ASMR and the number of deaths; **(C)** ASDR and the number of DALYs.

Regarding gender, the prevalence, mortality, and DALYs in 2021 were higher in females than in males, as were ASPR, ASMR, and ASDR. In terms of prevalence, both the number of cases and the ASPR for males and females exhibited an initial increase followed by a subsequent decrease. The peak in prevalence for both genders occurred between the ages of 55 and 59, while the peak in ASPR for both genders occurred between the ages of 75 and 79. Mortality for both males and females was slightly higher in the 0–4 age group, before reaching a peak at 80–84 years, with a gradual increase from ages 5–9. DALYs for both males and females peaked in the 0–4 age group. Furthermore, ASMR and ASDR in males increased steadily with age, both peaking in the 90–94 age group. In females, both ASMR and ASDR exhibited a marked upward trend with age, reaching their highest values in the 95+ age group (Figure 2).

**Figure 3:**
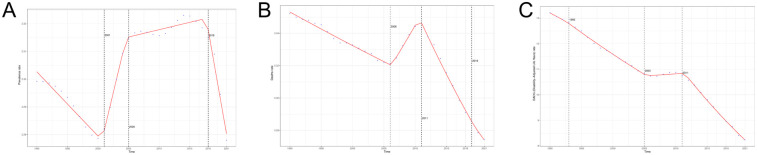
AAPC analysis of PAH **(A)** ASPR; **(B)** ASMR; **(C)** ASDR.

Based on the AAPC (Figure 3, Supplementary Table 3), we divided the period from 1990 to 2021 into four distinct phases to specifically examine the global trends in epidemiological indicators associated with PAH. Over the three decades, the overall change in the Age-Standardized Prevalence Rate (ASPR) for PAH was relatively steady, exhibiting a slight downward trend with minimal fluctuations (AAPC = −0.001; 95% CI: −0.001, −0.001). The most significant increase occurred between 2001 and 2005, while the most pronounced decrease was observed during 2018–2021. The ASPR for males demonstrated a generally upward trend (AAPC = 0.001; 95% CI: 0.001, 0.001), with the most notable increase occurring between 2000 and 2006, followed by a substantial decline during 2017–2021. The ASPR for females mirrored the overall trend, exhibiting a declining trajectory (AAPC = −0.002; 95% CI: −0.002, −0.003), with the greatest reduction occurring between 2018 and 2021. The Age-Standardized Mortality Rate (ASMR) for PAH showed an overall downward trend since 1990 (AAPC = −0.003; 95% CI: −0.002, −0.003), with a temporary rebound between 2006 and 2011. The most significant decline in ASMR was observed between 2011 and 2019. Both males (AAPC = −0.004; 95% CI: −0.003, −0.004) and females (AAPC = −0.002; 95% CI: −0.002, −0.002) exhibited similar trends, with both groups experiencing a decline, with the most substantial decrease in ASMR for males occurring between 2011 and 2019, and for females between 2011 and 2021.In terms of the Age-Standardized Death Rate (ASDR), significant reductions were observed both in the overall population (AAPC = −0.164; 95% CI: −0.161, −0.167) as well as in males (AAPC = −0.199; 95% CI: −0.196, −0.202) and females (AAPC = −0.128; 95% CI: −0.125, −0.131), with the most substantial decreases in ASDR occurring between 2011 and 2021 for both the overall population and males, and between 2013 and 2021 for females.

### Regional burden of PAH

At the regional level, the world is categorized into 21 regions based on a combination of geographic, economic, and cultural factors (2021; Table 1, Figure 1). Western Europe reported the highest Age-Standardized Prevalence Rate (ASPR) for PAH, at 3.56 per 100,000, while South Asia had the lowest at 1.71 per 100,000. East Asia had the highest absolute prevalence, with 42,486 individuals affected, whereas Oceania had the lowest, with only 196 individuals. From 1990 to 2021, all Global Burden of Disease (GBD) regions demonstrated an upward trend in the number of prevalent cases. Approximately half of the regions exhibited an increasing trend in the ASPR for PAH, with Western Sub-Saharan Africa showing the highest increase [EAPC = 0.98 (95% CI: 0.78, 1.18)], while Central Sub-Saharan Africa experienced the greatest decline [EAPC = −1.14 (95% CI: −1.30, −0.99)].North Africa and the Middle East reported the highest Age-Standardized Mortality Rate (ASMR) at 0.44 per 100,000, while Central Latin America had the lowest at 0.08 per 100,000. East Asia had the highest number of deaths, totaling 7,489 individuals, and Oceania recorded the fewest, with only 24 deaths. Overall, 80% of the GBD regions showed an increasing trend in the number of deaths. Excluding Central Asia, where the ASMR showed an upward trend, all other GBD regions experienced a decline, with Eastern Europe exhibiting the most pronounced decrease [EAPC = −3.78 (95% CI: −4.20, −3.36)].Regarding the Age-Standardized Death Rate (ASDR), North Africa and the Middle East had the highest rate at 14.81 per 100,000, while Central Latin America had the lowest at 3.00 per 100,000. East Asia recorded the highest number of Disability-Adjusted Life Years (DALYs) at 154,740, while Australasia had the fewest, with 1,433 DALYs. Over the past three decades, nearly two-thirds of the GBD regions experienced an upward trend in the number of DALYs. All regions exhibited a downward trend in ASDR, with Eastern Europe showing the largest decrease [EAPC = −3.99 (95% CI: −3.57, −4.41)].

### National burden of PAH

**Figure 4:**
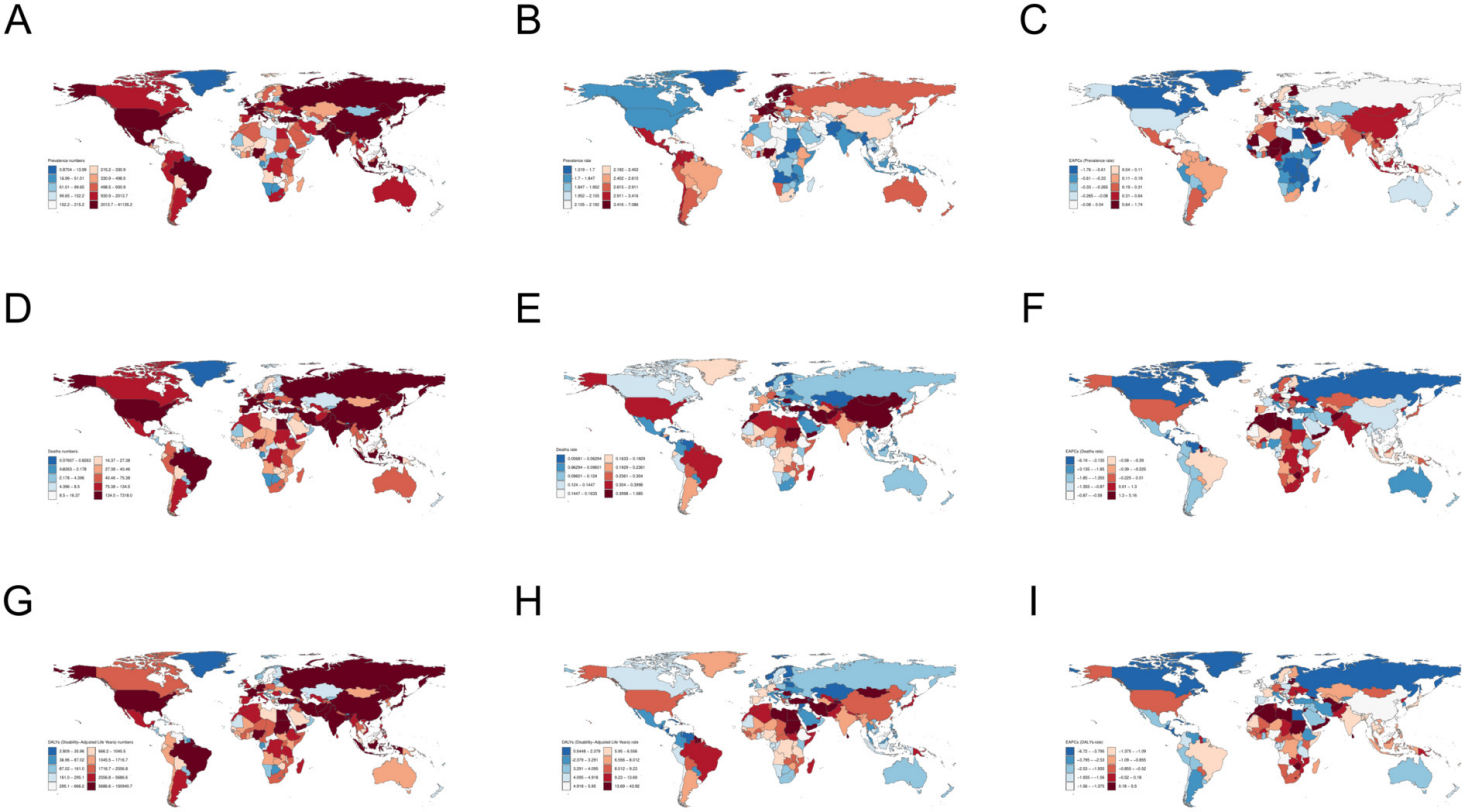
Global map of PAH **(A–C)** the number of prevalence, ASPR, and EAPC of ASPR; **(D–F)** the number of deaths, ASMR, EAPC of ASMR; **(G–I)** the number of DALYs, ASDR, EAPC of ASDR.

In 2021, the Swiss Confederation, Sweden, and the Netherlands exhibited the highest Age-Standardized Prevalence Rates (ASPR) among all countries, with values of 7.09 per 100,000 for Switzerland, 6.30 per 100,000 for Sweden, and 4.66 per 100,000 for the Netherlands, respectively. Nigeria (EAPC = 1.74), El Salvador (EAPC = 1.33), and Bangladesh (EAPC = 1.24) experienced the most significant increases in ASPR (Supplementary Table 2, Figure 4). Mongolia and Georgia reported the highest Age-Standardized Mortality Rates (ASMR), with 1.59 per 100,000 in Mongolia and 1.01 per 100,000 in Georgia. Tajikistan ranked third in ASMR, though with a rate below 1 per 100,000. Latvia (EAPC = 5.63), Taiwan (EAPC = 5.40), and Moldova (EAPC = 4.88) saw the largest increases in ASMR.

Mongolia, Mauritius, and Georgia had the highest Age-Standardized Death Rates (ASDR), with rates of 43.92 per 100,000 in Mongolia, 28.96 per 100,000 in Mauritius, and 27.83 per 100,000 in Georgia, respectively. Mauritius (EAPC = 5.50), Taiwan (EAPC = 4.76), and Georgia (EAPC = 3.48) also experienced the largest increases in ASDR. China, India, and the United States recorded the highest absolute numbers of prevalence, deaths, and Disability-Adjusted Life Years (DALYs) of any countries, with 41,135 cases, 7,317 deaths, and 150,940 DALYs in China; 23,741 cases, 2,611 deaths, and 94,736 DALYs in India; and 7,689 cases, 1,785 deaths, and 361,967 DALYs in the United States. Conversely, the countries with the lowest ASPR were Greenland (1.32 per 100,000), Pakistan (1.38 per 100,000), and Afghanistan (1.46 per 100,000). The largest decreases in ASPR were observed in the Arab Republic of Egypt (EAPC = −1.76), Rwanda (EAPC = −1.69), and Burundi (EAPC = −1.52). With the exception of Mongolia and Georgia, most countries and territories exhibited an ASMR of less than 1 per 100,000, with Moldova (0.01 per 100,000), Montenegro (0.03 per 100,000), and Slovenia (0.04 per 100,000) having the lowest ASMRs. Puerto Rico (EAPC = −6.64), Guatemala (EAPC = −5.93), and Costa Rica (EAPC = −5.69) experienced the most notable declines in ASMR. The countries with the lowest ASDR were Moldova (0.54 per 100,000), Slovenia (1.03 per 100,000), and Montenegro (1.05 per 100,000), respectively. The largest reductions in ASDR were observed in Puerto Rico (EAPC = −6.72), Greenland (EAPC = −5.50), and Guatemala (EAPC = −5.45). Tokelau, Niue, and Palau had the fewest instances of prevalence, deaths, and DALYs, with numbers falling below one.

### Trends in SDI and PAH disease burden

**Figure 5:**
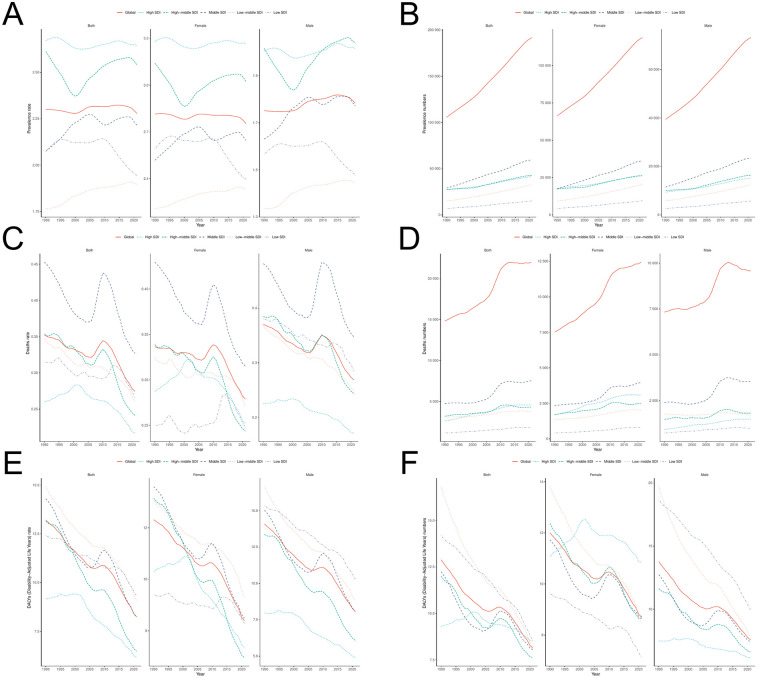
Time trend analysis of PAH **(A,B)** ASPR and the number of prevalence **(C,D)** ASMR and the number of deaths; **(E,F)** ASDR and the number of DALYs.

The GBD database classifies the world into five regions based on quintiles of the Socio-Demographic Index (SDI): High SDI, High-middle SDI, Middle SDI, Low-middle SDI, and Low SDI regions. From 1990 to 2021, the Age-Standardized Prevalence Rate (ASPR) in the Low-middle SDI and Middle SDI regions exhibited an increasing trend, while the ASPR in the remaining three regions demonstrated a declining trend. The number of prevalent cases of PAH increased across all five SDI regions (Figure 5). The High SDI region had the highest ASPR (2.64 per 100,000), while the Low-middle SDI region had the lowest ASPR (1.90 per 100,000). The Middle SDI region had the highest number of prevalent cases (59,666 individuals), whereas the Low SDI region recorded the lowest number (15,180 individuals) (Table 1). All five SDI regions showed a decreasing trend in the Age-Standardized Mortality Rate (ASMR), but the number of deaths increased in all regions. The Middle SDI region had the highest ASMR (0.33 per 100,000) and the highest number of deaths (7,548 individuals). In contrast, the High SDI region had the lowest ASMR (0.22 per 100,000), and the Low SDI region had the fewest deaths, totaling 1,781 individuals. In terms of Disability-Adjusted Life Years (DALYs) for PAH, all five regions exhibited a decreasing trend in the Age-Standardized Death Rate (ASDR). Both the Low SDI and High SDI regions showed an increase in the number of DALYs, while the remaining three regions experienced reductions compared to previous periods. The Low SDI region had the highest ASMR (9.30 per 100,000), while the High SDI region reported the lowest ASDR (6.16 per 100,000) and the lowest number of DALYs (93,181 individuals). The Middle SDI region had the highest number of DALYs, amounting to 197,170 individuals.

**Figure 6:**
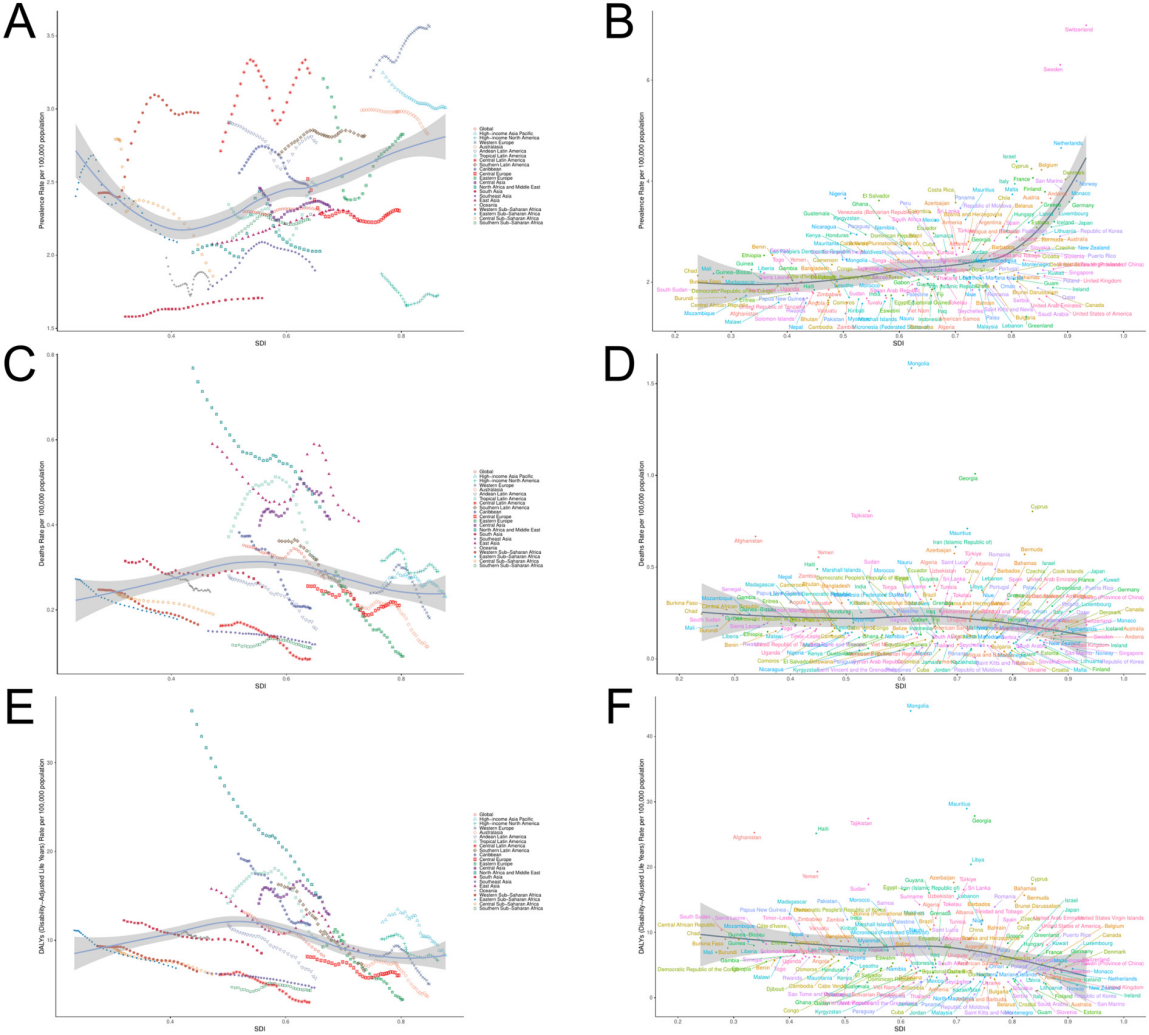
PAH burden and SDI trend analysis **(A)** ASPR and SDI analysis in 21 regions; **(B)** ASPR and SDI analysis of 204 countries/regions; **(C)** ASMR and SDI analysis in 21 regions; **(D)** ASMR and SDI analysis of 204 countries/regions; **(E)** ASDR and SDI analysis in 21 regions; **(F)** ASDR and SDI analysis of 204 countries/regions.

Over more than three decades, in 21 regions, the Age-Standardized Prevalence Rate (ASPR) exhibited an inverse “L” relationship with SDI levels, whereas the Age-Standardized Mortality Rate (ASMR) and Age-Standardized Death Rate (ASDR) demonstrated an “N” shaped relationship with SDI levels. In 204 countries or regions, ASPR showed an upward trend as SDI values increased, with a moderate positive correlation between ASPR and SDI levels. In contrast, ASMR and ASDR showed minimal fluctuation as SDI values increased (Figure 6).

### Decomposition analysis

**Figure 7:**
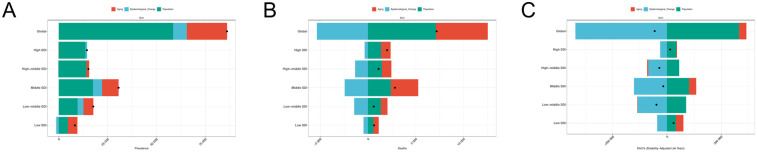
Decomposition analysis of PAH **(A)** the number of prevalence; **(B)** the number of deaths; **(C)** the number of DALYs.

We analyzed the impact of population growth, population aging, and epidemiological trends on the burden of PAH over three decades. Decomposition analyses revealed a growing trend in the global prevalence of PAH, with the most substantial increase occurring in the Middle-SDI region, followed by the Low-middle-SDI region (Figure 7). Population aging and population growth contributed 23.85% and 67.91%, respectively, to the increase in global prevalence, with these factors having a more significant effect on females than on males. The greatest contribution from population aging was observed in Low-SDI regions (59.29%), while the greatest contribution from population growth was seen in High-SDI regions (94.95%). Epidemiological trends accounted for 8.24% of the overall increase in prevalence, with a more pronounced effect on males than females. Low-middle-SDI regions contributed the most to the influence of epidemiological trends (16.62%), while Low-SDI regions showed the greatest negative impact of epidemiological trends (−16.20%).The number of deaths continued to rise globally, primarily driven by population growth (75.47%) and population aging (99.05%), with epidemiological trends serving as a mitigating factor (−74.51%), affecting males to a greater extent. The Low-middle-SDI region exhibited the highest contributions from population aging (116.43%), population growth (222.85%), and epidemiological trends (−239.28%).The number of Disability-Adjusted Life Years (DALYs) due to PAH has decreased over the past three decades, with population aging (−59.66%), population growth (−585.65%), and epidemiological trends (745.31%) all contributing to this decline, with a greater impact on females. The Middle-SDI region showed significant contributions to the reduction in DALYs due to population aging (−191.10%), population growth (−590.79%), and epidemiological trends (881.89%).

### Analysis of health inequalities

**Figure 8:**
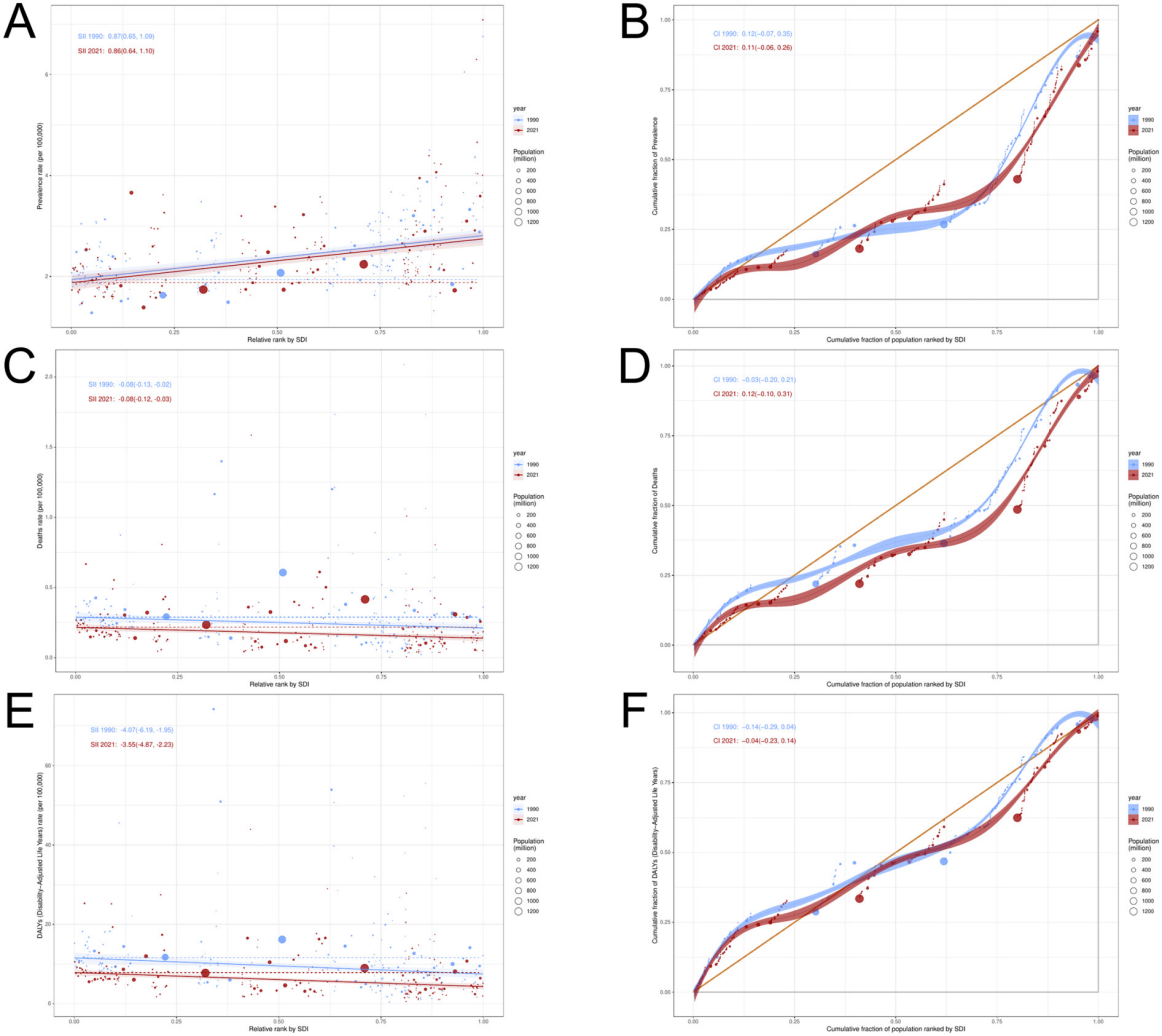
Health inequality analysis of PAH **(A,B)** ASPR; **(C,D)** ASMR; **(E,F)** ASDR.

The results of the slope index (SII) analysis (Figure 8) indicated that the SII value for the Age-Standardized Prevalence Rate (ASPR) in PAH decreased slightly from 0.87 (95% CI: 0.65, 1.09) in 1990 to 0.86 (95% CI: 0.64, 1.10) in 2021. This suggests that countries and regions with higher SDI continue to disproportionately bear a greater burden of ASPR in PAH. The SII value for the Age-Standardized Mortality Rate (ASMR) in 2021 [−0.08 (95% CI: −0.13, −0.02)] showed no significant change from the SII value in 1990 [−0.08 (95% CI: −0.13, −0.02)]. In contrast, the SII for the Age-Standardized Death Rate (ASDR) decreased from −4.07 (95% CI: −1.95, −6.19) in 1990 to −3.55 (95% CI: −2.23, −4.87), indicating that countries and regions with lower SDI continue to disproportionately bear a heavier burden of ASMR and ASDR in PAH.

The Confidence Intervals (CIs) of the concentration index revealed that both the prevalence (1990: 0.12; 2021: 0.11) and Disability-Adjusted Life Years (DALY) rates (1990: −0.14; 2021: −0.04) for PAH exhibited varying degrees of decline from 1990 to 2021. In contrast, the mortality rate (1990: −0.03; 2021: 0.12) for PAH showed an increasing trend. These results indicate that both the absolute and relative inequality of Age-Standardized Prevalence Rate (ASPR) and Age-Standardized Death Rate (ASDR) for PAH improved from 1990 to 2021, while the absolute inequality of Age-Standardized Mortality Rate (ASMR) showed no significant improvement, and the relative inequality remained unchanged.

### Frontier analysis

**Figure 9:**
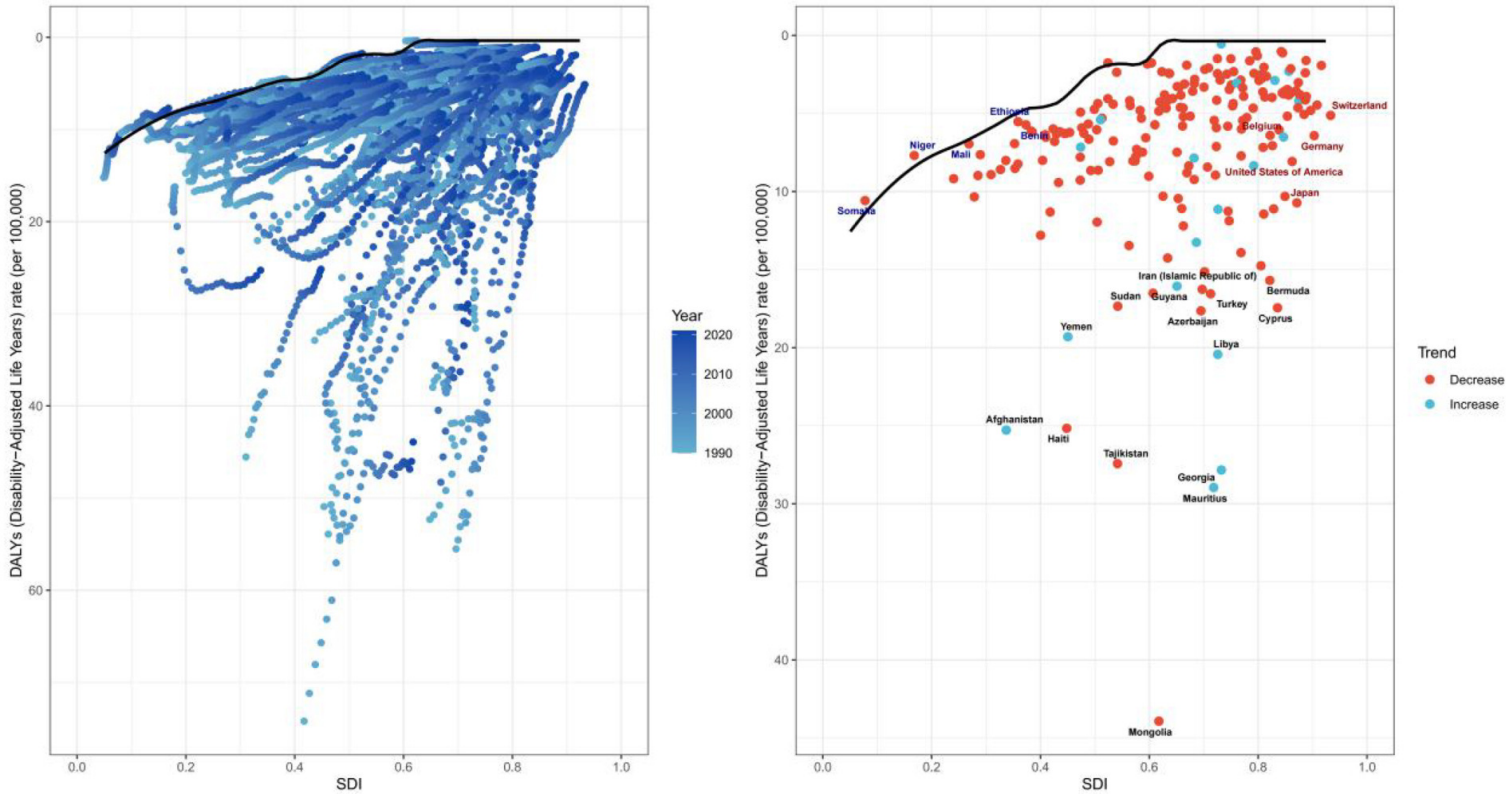
Frontier analysis of PAH.

We conducted a frontier analysis of the Age-Standardized Death Rate (ASDR) for PAH (Figure 9). The results of the analysis revealed that the top 15 countries/regions with the largest actual differences in potential improvements in ASDR (with effective differences ranging from 15.33 to 43.46) include Mongolia, Mauritius, Georgia, Tajikistan, Haiti, Libya, Afghanistan, Azerbaijan, Cyprus, Turkey, Iran, Guyana, Sudan, Yemen, and Bermuda. Conversely, the 10 countries or regions with the smallest differences between their ASDR and the frontier line, representing those with lower SDIs, include Somalia, Niger, Mali, Nicaragua, and Guatemala, while those with higher SDIs include Venezuela, Kyrgyzstan, Moldova, Montenegro, and Slovenia.

Frontier analyses reveal significant potential for reducing the PAH disease burden across countries and regions, with effective differences generally increasing as sociodemographic factors evolve. This suggests that countries or regions with higher SDIs have greater potential for improvement in reducing the burden of PAH.

### APC model

The Age-Related Effect (ARC) is a commonly used concept in epidemiology and social sciences, referring to the influence of age on a particular phenomenon (e.g., disease incidence, mortality, behavioral habits, etc.). This effect is typically assessed by analyzing the health status, behavioral patterns, and exposure to risk factors among individuals of different ages, in order to understand the role of age in shaping a given outcome.

**Figure 10:**
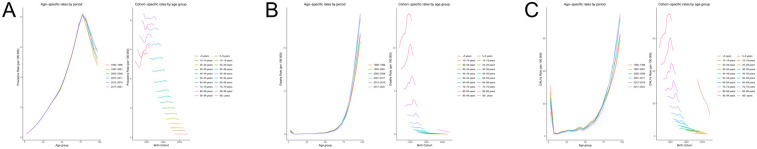
Age specific effects of PAH **(A)** ASPR; **(B)** ASMR; **(C)** ASDR.

Between 1992 and 2021 (Figure 10), the Age-Standardized Prevalence Rate (ASPR) for PAH initially increased and then decreased with age, the Age-Standardized Mortality Rate (ASMR) increased significantly with age, and the Age-Standardized Death Rate (ASDR) showed a pattern of first decreasing and then increasing with age. Birth cohort data revealed that the ASPR in the 65+ age group initially decreased and then increased over time, remaining relatively stable in the other age groups. The ASMR and ASDR in the 70+ age group generally exhibited a trend of increase followed by a decrease. Notably, the ASDR in the 0–4 year age group demonstrated a significant downward trend over time.

**Figure 11:**
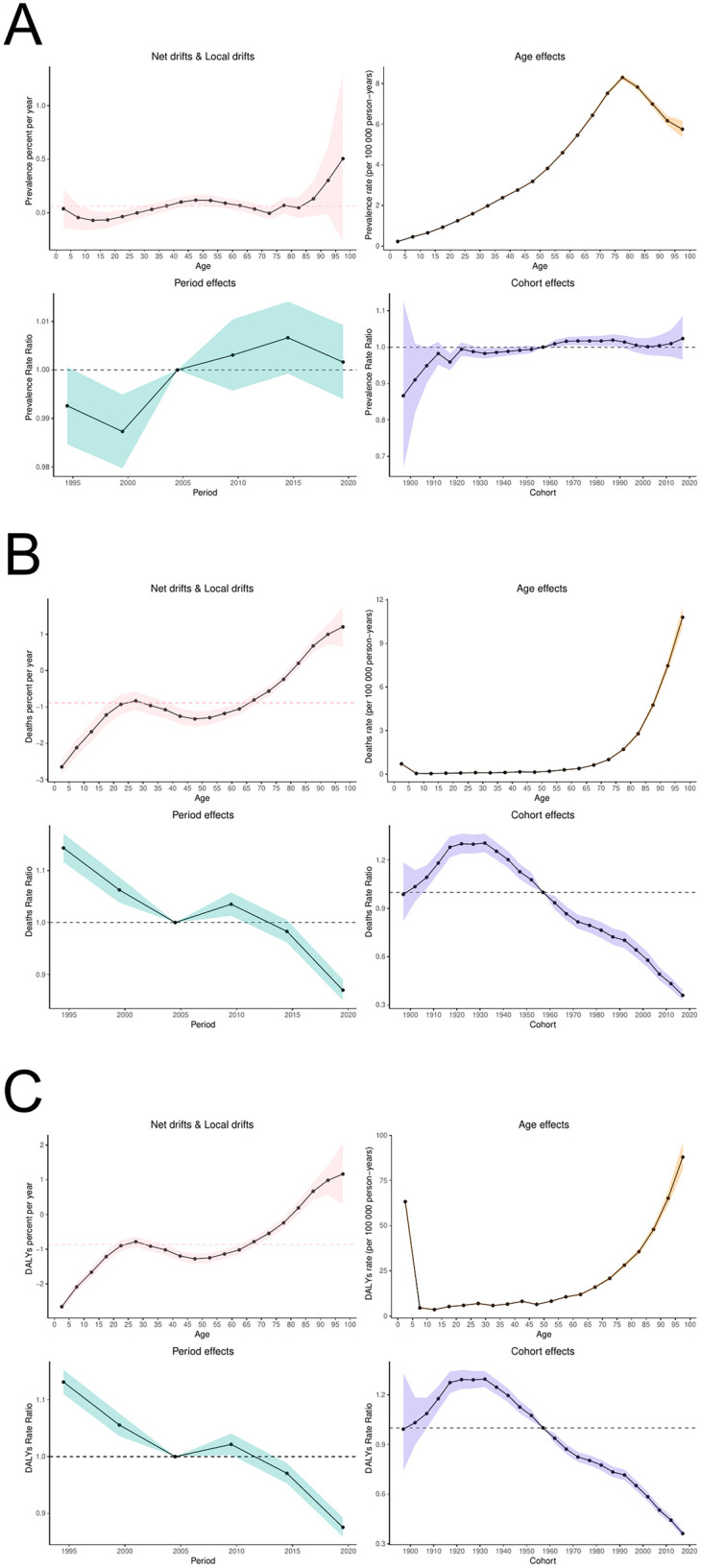
APC model of PAH **(A)** prevalence rate; **(B)** death rate; **(C)** DALYs rate.

The overall net drift in global PAH prevalence rate, death rate, and DALY rates was 0.060% (95% CI: 0.030, 0.092), −0.889% (95% CI: −0.977, −0.800), and −0.860% (95% CI: −0.926, −0.794), respectively, indicating an increase in prevalence over the study period, alongside a decline in both death rate and DALY rate (Figure 11). The prevalence rate was higher in men [0.174 (95% CI: 0.115, 0.233)] compared to women [0.006 (95% CI: −0.032, 0.044)]; conversely, the death rate [−1.080 (95% CI: −1.214, −0.945)] and DALY rate [−1.048 (95% CI: −1.127, −0.969)] showed more substantial improvements in men than in women [−0.742 (95% CI: −0.861, −0.623)] and [−0.717 (95% CI: −0.784, −0.650)], respectively. Localized drift accounted for additional age-related changes in epidemiologically relevant trends. For PAH prevalence, localized drift was negative for the 5–29 and 70–74 age groups, suggesting an improvement in prevalence within these populations. For the death rate and DALY rate, localized drift values were negative for the under-80 age group, indicating improved outcomes in terms of both risk of death and DALY burden in this group over the previous period.

The age effects on prevalence rate, death rate, and DALY rate for PAH exhibited distinct trends. The prevalence rate began to rise with age, peaking at the 75–79 years age group, and then declined thereafter. The death rate progressively increased with age, while the DALY rate was slightly higher in the 0–4 years age group, after which it increased significantly with age, starting from its lowest value in the 5–9 years age group.Time effects revealed a gradual increase in the prevalence of PAH throughout the study period, while both mortality and DALY rates showed considerable improvement. Cohort effects indicated an increased risk of PAH prevalence for cohorts born after 1957, with this risk intensifying over time. In terms of risk of death and DALY, the birth cohort born after 1957 showed both improvement and an increasing trend over time.

### BAPC model

**Figure 12:**
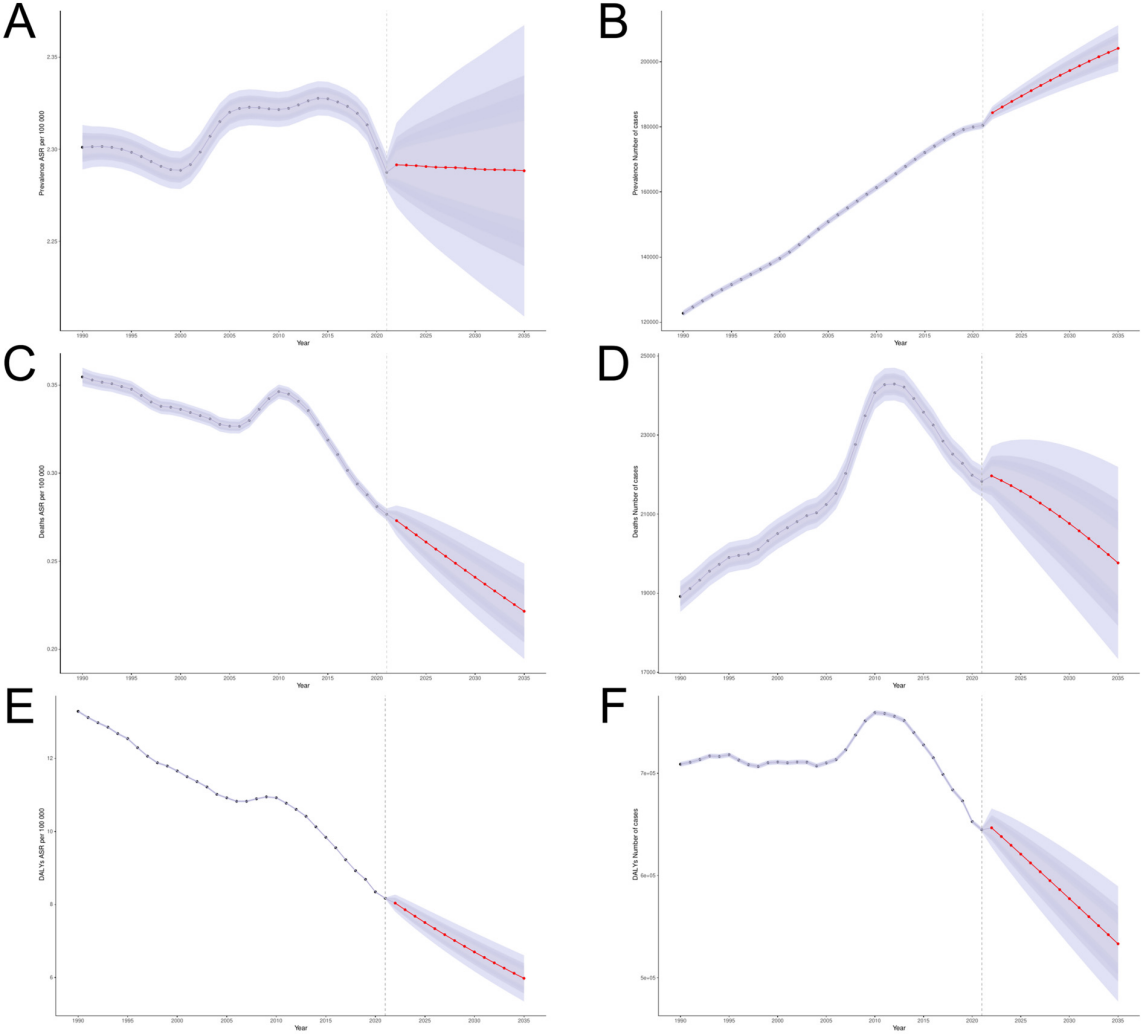
BAPC model of PAH **(A,B)** ASPR and the number of prevalence; **(C,D)** ASMR and the number of deaths; **(E,F)** ASDR and the number of DALYs.

**Figure 13:**
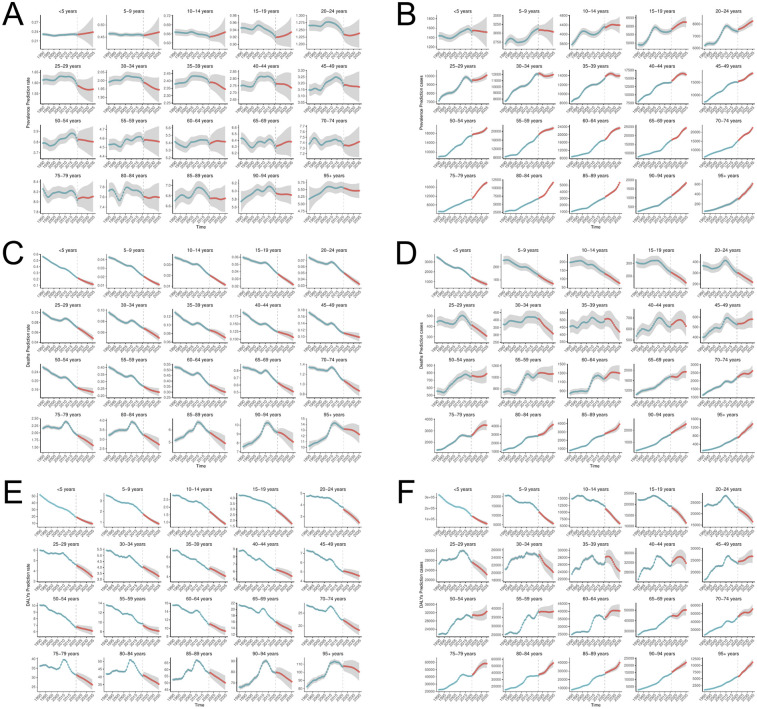
BAPC model **(A,B)** ASPR and and the number of prevalence; **(C,D)** ASMR and the number of deaths; **(E,F)** ASDR and the number of DALYs.

The BAPC analysis projected the global disease burden of PAH for 2035, with further subgroup analysis by gender and age groups. The number of PAH cases is expected to rise to 204,148 people (a 13.09% increase) by 2035, with a corresponding increase in the Age-Standardized Prevalence Rate (ASPR) to 2.288 per 100,000 (Figure 12). Across age groups (Figure 13), the ASPR is expected to show a slight downward trend in the 25–59 age group, a slight upward trend in the 0–24 and 60–79 age groups, and a relatively stable trend in the remaining age groups. The number of prevalence cases is projected to increase in all age groups over the next 10 years. The number of deaths is projected to decrease to 19,767 people (−9.45%), with the Age-Standardized Mortality Rate (ASMR) expected to reach 0.221 per 100,000. The ASMR is projected to show a significant downward trend across all age groups, with a notable decrease in deaths in the 0–39 age group and an increase in the 40+ age group. The number of Disability-Adjusted Life Years (DALYs) is projected to decrease to 533,159 people by 2035 (−17.30%), with the Age-Standardized Death Rate (ASDR) expected to decrease to 5.98 per 100,000. ASDR is projected to show a significant decline across all age groups, with DALYs decreasing in the 0–39 age group and increasing in the 40+ age group.

## Discussion

PAH, a progressive and incurable chronic disease, severely impacts both the life expectancy and quality of life of affected individuals (34). Despite its relative rarity, it remains a significant challenge and one of the major contributors to the socioeconomic burden. In this study, we utilized the GBD 2021 database to gather epidemiological data on PAH and employed a variety of analytical methods to examine the disease burden in a comprehensive, multilevel manner.

The slight decrease in the Age-Standardized Prevalence Rate (ASPR), Age-Standardized Mortality Rate (ASMR), and Age-Standardized Death Rate (ASDR) for PAH from 1990 to 2021 can be attributed to several factors. First, advancements in medical technology, such as echocardiography (35) and right heart catheterization (36), have significantly improved early detection of PAH, reducing underdiagnosis and enhancing opportunities for targeted treatment and management (37). Second, clinical trials exploring drug combinations for PAH have been pivotal in decreasing the risk of clinical deterioration and associated mortality, as well as in delaying disease progression (38–41). Additionally, the increasingly refined PAH risk assessment and prognostic management strategies worldwide have likely contributed to these improvements (42). For instance, in 2012, researchers developed the REVEAL 1.0 risk score calculator, which assesses early and long-term PAH management based on data from the WHO cohort (43). In 2019, the tool was updated to REVEAL 2.0, incorporating new data on predictors of disease progression and mortality, which allowed for more accurate patient risk stratification, helping clinicians identify high-risk individuals early and enhance clinical decision-making to improve prognosis (44, 45). Furthermore, the 2022 update of the European Society of Cardiology/European Respiratory Society (ESC/ERS) guidelines comprehensively revised the definition, diagnosis, and treatment of PAH, providing a critical framework for subsequent treatment and management strategies (10).

The significant increase in the number of prevalent cases (81.46%) and deaths (48.36%) from PAH compared to 1990 highlights a growing global burden of the disease, particularly in East Asia, including China and India. These regions, home to about 25% of the global population, naturally rank among the highest in terms of prevalence, deaths, and Disability-Adjusted Life Years (DALYs) due to PAH. As a result, it is crucial for these areas to strengthen policy coordination and allocate more resources to address the increasing disease burden of PAH. This can include enhancing public health campaigns, improving healthcare infrastructure, and ensuring better access to treatments. However, despite PAH being treatable with combination therapies, many developing or low-income countries face challenges in affording high drug costs and in receiving sufficient governmental subsidies, which often results in patients receiving only monotherapy. This greatly increases the risk of disease progression and death (46). Moreover, the global disparity in PAH burden is stark, with a 100-fold difference in the number of cases between the regions with the highest and lowest burdens. For instance, Oceania and Australia, which have well-developed healthcare systems, report the lowest disease burden for PAH. The combination of high-income status, advanced medical technology, specialized healthcare staff, and well-established healthcare policies like Australia's Pharmaceutical Benefits Scheme (PBS) (47) ensures early diagnosis and effective treatment, thus reducing DALY rates and mortality. These regional disparities also reflect health inequalities, where regions with higher Socio-Demographic Index (SDI) scores tend to bear a disproportionately high burden of the disease, while regions with lower SDIs face higher risks of death and DALYs. To address this, there is an urgent need for targeted interventions in low-income regions. These could include more rational allocation of healthcare resources, greater investment in healthcare infrastructure, and enhanced efforts to improve diagnosis and treatment capacity, particularly in areas with fewer financial resources (48).

Notably, the global trend in Disability-Adjusted Life Years (DALYs) for Pulmonary Arterial Hypertension (PAH) diverges from the patterns typically seen in other diseases. This anomaly can be attributed to a range of international initiatives focused on early detection, treatment, and heightened awareness of the disease. Such efforts, coupled with advancements in social mobilization and monitoring strategies, have played a pivotal role in alleviating the overall burden of PAH.

Our findings indicate that the burden of Pulmonary Arterial Hypertension (PAH) is more pronounced in women than in men, suggesting the need for more targeted and effective prevention and management strategies for females. Female susceptibility to PAH may be intricately linked to sex hormones. Several studies have demonstrated (49, 50) that gender differences are pivotal in the pathophysiology of pulmonary arterial hypertension. On one hand, sex hormones such as estrogen may influence both the physiological and pathological processes of the pulmonary vasculature, thereby affecting the pathogenesis of PAH in women. It has been suggested that estrogen could facilitate the proliferation and remodeling of the pulmonary vascular endothelium, thereby heightening the risk of PAH in women (51). On the other hand, the occurrence of PAH during pregnancy and/or postpartum further underscores the influence of sex hormones on the disease in women (52). Despite the higher prevalence of pulmonary hypertension in female patients, numerous experimental studies have indicated that female animals tend to exhibit a better prognosis, a phenomenon often referred to as the “estrogen paradox” of pulmonary hypertension. However, reconciling and addressing this paradox remains an ongoing challenge (53). The age-period-cohort effects revealed that the burden of ASPR and ASMR in PAH predominantly affects the elderly, with the risk of morbidity and mortality being generally positively correlated with age. This may be reflective of the global issue of aging populations. Both the time and cohort effects suggest that medical advancements over the past three decades have significantly enhanced the accuracy of PAH diagnoses, leading to a relative reduction in the risk of death and disability attributable to the disease. While the age effect of DALY rates shows a positive correlation with age in individuals over 5 years of age, the slightly elevated DALY rate of PAH in the under-5 age group warrants attention. This could be related to insufficient public awareness regarding health insurance coverage, immunization rates, and preventive treatment education. Therefore, it is imperative for relevant national authorities to enhance public awareness campaigns, broaden vaccination coverage, and allocate medical resources and drug management more equitably (54).

We applied frontier analysis to assess the potential room for improvement in PAH in 204 countries and territories; traditional linear models focus on existing relationships between variables and may ignore this potential for improvement because they do not take into account achievable benchmarks related to the level of SDI (28). Some countries with low SDI (e.g., Somalia, Niger) exist as frontier countries, and despite limited economic conditions and social resources, they have shown surprising results that should serve as a model for optimizing health outcomes in resource-poor settings (55). In contrast, some countries with high SDIs (e.g., Mauritius) have performed poorly in managing the PAH disease burden and still have a high potential for improvement. Excluding geographic location, these countries and regions still need to make active efforts in environmental management, universal access to health services, and drug resource allocation to further optimize and improve social policies. At the same time, there should be appropriate exchanges between countries to clarify the drivers of disease burden reduction in low SDI countries and the reasons for the large effective differences in high SDI countries, so as to minimize the differences in disease burden between countries.

Although the BAPC model predicts a declining trend in the global burden of death and DALYs burden of PAH, the number of prevalence for PAH and ASPR will increase in 2035, suggesting that new challenges remain in the control and management of PAH. Our decomposition analysis showed that the increase in the prevalence of PAH over the past three decades was mainly attributed to population growth and population aging, and thus population expansion, including population growth and population aging, may also be responsible for the projected increase in the prevalence of PAH by 2035. It has been reported (56) that the United Nations states that the global population will continue to grow to 9.7 billion by 2050; the percentage of the global population over 60 will grow to 22% by 2050. Therefore, countries around the world should pay attention to this demographic issue and improve their healthcare systems to adequately cope with this demographic change. It is important to note that the projected estimates for 2035 should be interpreted with caution, as both the sparseness of the data and the small variations exhibited by the ASR can create significant uncertainty in the estimates of mortality and DALY rates. Nonetheless, it still highlights the efforts that have been made to prevent PAH and reduce DALY (57).

This study has several limitations. First, while the GBD data encompasses a broad array of sources, some regions may not have accurately captured gender and temporal variations, potentially leading to biased results, particularly in low- and middle-income countries. Second, the analysis may have underestimated the burden of PAH. Since PAH diagnosis relies on imaging and invasive hemodynamic assessments, populations in poorer regions often face limited access to detection and treatment, which may result in the true prevalence of the disease being underreported (58). Third, differences in diagnostic criteria and practices across regions may affect the consistency and comparability of the data. Finally, the GBD database only adjusts for certain covariates, meaning unaccounted factors such as dietary habits, lifestyle, medication use, and metabolic and physiological changes could influence the results (59). These issues warrant further consideration in future iterations of the GBD framework.

## Summary

In summary, pulmonary arterial hypertension (PAH), a rare and fatal disease, represents a substantial global health burden and will continue to present significant challenges in terms of control and management in the future. To mitigate this burden, it is crucial to implement tailored interventions that consider gender, age, regional disparities, and levels of economic development. Furthermore, a more rational allocation of healthcare resources is necessary to enhance individualized healthcare systems and address the unique healthcare needs of each country.

## Data Availability

The original contributions presented in the study are included in the article/Supplementary Material, further inquiries can be directed to the corresponding author.
